# Stop, think, reflect, realize—first‐time mothers’ views on taking part in longitudinal maternal health research

**DOI:** 10.1111/hex.12861

**Published:** 2019-02-21

**Authors:** Deirdre Daly, Margaret Carroll, Monalisa Barros, Cecily Begley

**Affiliations:** ^1^ School of Nursing and Midwifery Trinity College Dublin Dublin Ireland; ^2^ Departamento de Ciencias Naturais Universidade Estadual do Sudoeste da Bahia Vitoria Da Conquista BA Brazil; ^3^ Sahlgrenska Academy Institute of Health and Care Sciences University of Gothenburg Gothenburg Sweden

**Keywords:** cohort studies, maternal health, maternal morbidity, postpartum period, surveys and questionnaires, women's views

## Abstract

**Background:**

Longitudinal cohort studies gather large amounts of data over time, often without direct benefit to participants. A positive experience may encourage retention in the study, and participants may benefit in unanticipated ways.

**Objective:**

To explore first‐time mothers’ experiences of taking part in a longitudinal cohort study and completing self‐administered surveys during pregnancy and at 3, 6, 9 and 12 months’ postpartum.

**Design:**

Content analysis of comments written by participants in the **M**aternal health **A**nd **M**aternal **M**orbidity in **I**reland study's five self‐completion surveys, a multisite cohort study exploring women's health and health problems during and after pregnancy. This paper focuses on what women wrote about taking part in the research. Ethical approval was granted by the site hospitals and university.

**Setting and participants:**

A total of 2174 women were recruited from two maternity hospitals in Ireland between 2012 and 2015.

**Findings:**

A total of 1000 comments were made in the five surveys. Antenatally, barriers related to surveys being long and questions being intimate. Postpartum, barriers related to being busy with life as first‐time mothers. Benefits gained included gaining access to information, taking time to reflect, stopping to think and being prompted to seek help. Survey questions alone were described as valuable sources of information.

**Discussion and conclusions:**

Findings suggest that survey research can “give back” to women by being a source of information and a trigger to seek professional help, even while asking sensitive questions. Understanding this can help researchers construct surveys to maximize benefits, real and potential, for participants.

## 1 BACKGROUND

Taking part in longitudinal cohort studies, research which gathers data from the same subjects repeatedly over a period of time, requires considerable commitment from pregnant and postpartum women. While a positive experience may encourage participants to remain in the study, a negative one may lead to attrition, or even be harmful or unethical. Longitudinal cohort studies with large populations have multiple benefits, including being able to plot prevalence at different time points, study temporal changes and associations with a range of factors. As well as addressing current research questions^1^ and informing policy,^2^, ^3^, ^4^ participants can also be a resource for the future.^5^ While cohort studies can fulfil the interests of researchers, policy makers and clinicians,^1^ participants are seldom listed as beneficiaries. Studies have shown that some pregnant women take part in research, by completing questionnaires^6^ or enrolling in randomized trials,^7^ for altruistic reasons.^6^, ^7^ For some, taking part in research enhanced their personal experience or made them feel special while others felt it had the potential to be a negative experience.^6^ Mein et al^8^ found that, rather than being wholly motivated by altruism, research participants in their longitudinal study on the effect of social gradients on health were motivated by the perceived benefits of taking part, especially with the information and care received during medical examinations, and the sense of loyalty and membership associated with participating. Participants in longitudinal cohort studies may be asked to complete questionnaires, interviews and undergo a variety of tests over several months or years, often without any direct obvious benefit to them personally. However, participants may benefit, or perceive they benefit, in unforeseen ways. The most common reasons pregnant women gave for joining a South African birth cohort study were the belief that the study would improve their or their child's health, to receive better health care through participation, or because the study was important to friends’ or family's health.^9^ Women were recruited from two primary health care clinics serving different populations, a mixed‐race population and a black African population, and the location was described as having a high burden of childhood disease and risk factors for childhood illness. Reasons for staying in the study included the belief that their child's health was better for being in the study, and the opportunity to learn.^9^

## 2 THE MAMMI STUDY

The MAMMI (**M**aternal health **A**nd **M**aternal **M**orbidity in **I**reland) study is a multisite multistrand cohort study exploring the health and health problems experienced by 3048 first‐time mothers recruited from three maternity hospitals in Ireland between January 2012 and March 2017. Each strand focuses on one health problem, that is, urinary incontinence (leaking urine when you do not mean to), anal incontinence (passing wind or faeces [stools] when you do not mean to), pelvic girdle pain, sexual health, mental health and intimate partner violence. Recruitment has closed in all three sites and postpartum follow‐up is ongoing in one site. This paper presents data from 2174 women recruited from two of these sites, from women who completed one, several or all of the five surveys, up to 12 months postpartum. The study's aims are as follows: (a) to describe the existence, extent and prevalence of morbidity during pregnancy and the first year postpartum in nulliparous women; (b) determine risk factors associated with morbidities; (c) gain an in‐depth understanding of the impact of childbirth, motherhood and any related morbidity on women's health and health needs, and (d) identify women's health‐service and self‐help seeking behaviour. The combined data will be used to design future studies that test interventions for modifiable factors, for example, prevent or treat urinary incontinence, with separate cohorts of women.

### 2.1 Design

The MAMMI study is a mixed methods study incorporating self‐completion surveys administered at five time points, data collection from consenting women's maternity records and one‐to‐one interviews with women experiencing a specific morbidity, for example, pelvic girdle pain, sexual health problems. It was established as an exploratory sequential design, and each strand was designed to allow the primary focus to be on either the quantitative or qualitative component. This paper is an in‐depth content analysis of the comments first‐time mothers wrote in the five surveys about their experiences of taking part in the research.

### 2.2 Data collection

Permission was gained to modify the surveys used in the Australian Maternal Health Study (https://www.mcri.edu.au/research/projects/maternal-health-study) for use in Ireland. All core content was retained, and the terms used to describe the health and maternity services were changed to suit Ireland. The first antenatal survey asks women about their general health before pregnancy, and all surveys include questions, organized in separate sections (strands), on urinary incontinence, anal incontinence, pain, sexual and mental health issues. Postpartum surveys also include questions on intimate partner violence. Each section provides brief information on sources of help about that specific health issue and space to comment. The final page of each survey has the heading “Comments. If you wish to write any further comments please do so on this page. Thank you.” Surveys can be viewed on the study's website (http://www.mammi.i.e/surveys.php).

This paper presents data from 2174 women recruited from two maternity hospital sites, one large (>8500 births per annum) and one medium (>3000 births per annum) in Ireland. The study methods, described previously,^10^ are outlined below.

### 2.3 Eligibility

Eligible women were aged 18 years or over and able to understand English sufficiently to complete the surveys. Women who were recruited to the study and completed the first antenatal survey were excluded from follow‐up postpartum if they experienced a miscarriage, their baby was ill in a neonatal unit or had died.

### 2.4 Recruitment

At their first hospital booking visit, the midwife offered eligible women the study information pack which included a letter of introduction, an information booklet outlining the purpose of the research, two copies of the consent form (one to be retained by the woman), the first (antenatal) survey and a freepost addressed envelope for returning the survey and consent form. The midwife gained women's consent to being contacted by the researcher and, 1 to 2 weeks’ later, the researcher phoned each woman to answer questions and ascertain if she wished to take part. Women were regarded as recruited when the signed consent form and completed antenatal survey were received. Women self‐completed the surveys at home and returned them in freepost envelopes provided. No incentive was offered.

### 2.5 Postpartum follow‐up

Postpartum surveys were posted to women's home address 3 weeks in advance of when they were due to be completed. If a particular postpartum survey was not returned when it was due, the woman received three reminders over the following 2 months; telephone reminder, text reminder and, finally, the relevant postpartum survey was reposted. No further contact was made, and no follow‐up was made to women who indicated that they wished to withdraw from the study.

### 2.6 Participants

A total of 2174 participants were recruited from both sites. Retention percentages, based on the number of participants eligible for follow‐up at each time point, were as follows: 87.1% (n = 1771/2034) at 3 months; 84.9% (n = 1641/1933) at 6 months; 80.4% (n = 1540/1915) at 9 months and 78.1% (n = 1437/1840) at 12 months postpartum. The final sample of 1437 women retained at 12 months postpartum represents 66.1% of the 2174 women recruited and 70.6% of women eligible for 3‐month postpartum follow‐up.

### 2.7 Ethical considerations

Ethical approval was granted by the university's and site hospitals’ ethics committees, and all participating women gave written consent.

### 2.8 Data analysis

We used a conventional approach^11^ to analyse the content, that is, each participant's data were entered into one row, coded, and categories and themes identified. Data were analysed independently by MB and DD, then interpretations, codes and themes were discussed and agreed. Participants’ verbatim quotations from the surveys were identified to illustrate the themes that emerged. Microsoft Excel 2013^®^ software was used to manage the data.

## 3 FINDINGS

**Figure 1:**
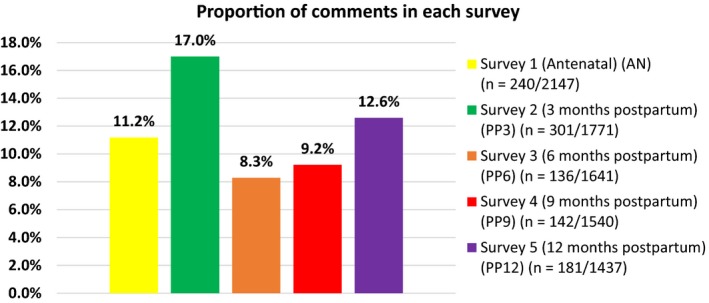
Proportion of comments in each survey

**Figure 2:**
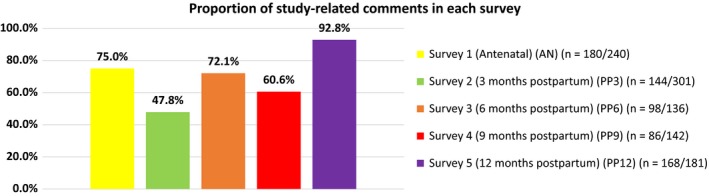
Proportion of study‐related comments in each survey

Characteristics of the 2174 women recruited are presented in Table S1, and are compared to local^12^ or national data,^13^, ^14^, ^15^ when available. Recruited women are broadly representative of women living in Ireland in 2011 in terms of nationality. Women's relationship status also reflects the status of women birthing in Ireland in 2013. Women aged 18‐24 years are under‐represented and women aged 30‐39 years are over‐represented compared to the age profile of women birthing in Ireland in 2013. Differences in how national data are reported prevents further comparison. The characteristics of the women who commented in each survey are presented in Table 1. The majority of comments were from women of Irish or any other white background but the proportion of comments, by age group, highest education attainment and relationship status at the time of completing each survey, differed minimally across surveys. However, fewer women with no formal or lower secondary education commented in any survey. The number and proportion of comments written on the last page in each survey are shown in Figure 1. Some comments were single sentences and others were a full A4 page of text. Many women used this page to give more details on their experiences of becoming a first‐time mother, their family circumstances, and the care received, while others commented on taking part in the study. Three themes emerged: “barriers and benefits of taking part in the research”; “quality of care received” and “the pregnancy and birth experience.” The proportion of comments in each survey that mentioned some aspect of the study are presented in Figure 2 and include those that simply stated comments like “Thank you for doing this research (study).” The findings presented here are based on the comments that specified some barrier to or benefit of taking part in the study.

**Table 1:** Characteristics of women who commented in each survey

	MAMMI study (n = 2174)		Survey 1 Antenatal (AN) n = 240		Survey 2 3 months postpartum (PP3) n = 301		Survey 3 6 months postpartum (PP6) n = 136		Survey 4 9 months postpartum (PP9) n = 142		Survey 5 12 months postpartum (PP12) n = 181	
	n	%	n	%	n	%	n	%	n	%	n	%
Nationality												
Irish (including Irish Traveller, n = 2)	1559	71.7	182	75.8	232	77.1	107	78.7	113	79.6	155	85.6
African	23	1.1	4	1.7	1	0.3	1	0.7	0	0.0	0	0.0
Chinese	11	0.5	3	1.3	1	0.3	1	0.7	0	0.0	0	0.0
Any other white background	506	23.3	46	19.2	58	19.3	24	17.6	28	19.7	24	13.3
Any other Asian background	42	1.9	3	1.3	3	1.0	3	2.2	1	0.7	0	0.0
Other, including mixed background	23	1.0	0	0.0	5	1.7	0	0.0	0	0.0	2	1.1
Not stated	10	0.5	2	0.8	1	0.3	0	0.0	0	0.0	0	0.0
Age group												
18 to 24 y	177	8.1	25	10.4	22	7.3	11	8.1	8	5.6	14	7.7
25 to 29 y	471	21.7	51	21.3	54	17.9	24	17.6	28	19.7	38	21.0
30 to 34 y	941	43.3	97	40.4	113	37.5	55	40.4	59	41.5	74	40.9
35 to 39 y	484	22.3	52	21.7	87	28.9	38	27.9	38	26.8	46	25.4
Over 40 y	94	4.3	14	5.8	24	8.0	7	5.1	8	5.6	8	4.4
Not stated	7	0.3	1	0.4	1	0.3	1	0.7	1	0.7	1	0.6
Relationship status^a^												
Married	1312	60.3	138	57.5	196	65.1	83	61.0	92	64.8	122	67.4
Living with partner	575	27.3	70	29.2	89	29.6	41	30.1	37	26.1	46	25.4
In a relationship—not living together	160	7.4	16	6.7	7	2.3	6	4.4	6	4.2	5	2.8
Single (including divorced, separated or widowed, n = 5)	84	3.9	12	5.0	9	3.0	5	3.7	7	4.9	7	3.9
Other	17	0.8	1	0.4	0	0.0	0	0.0	0	0.0	0	0.0
Not stated	6	0.3	3	1.3	0	0.0	1	0.7	0	0.0	1	0.6
Highest education attainment												
No formal—lower secondary	51	2.4	7	2.9	5	1.7	2	1.5	2	1.4	1	0.6
Upper secondary, apprenticeship/vocational	488	22.5	50	20.8	63	20.9	23	16.9	29	20.4	35	19.3
National certificate/Diploma, Institute of Technology or equivalent	205	9.4	20	8.3	35	11.6	17	12.5	12	8.5	21	11.6
Primary degree/professional qualification of degree status/postgraduate certificate or diploma	952	43.8	94	39.2	124	41.2	62	45.6	62	43.7	88	48.6
Postgraduate degree/PhD	464	21.3	68	28.3	72	23.9	32	23.5	36	25.4	36	19.9
Not stated	14	0.6	1	0.4	2	0.7	0	0.0	1	0.7	0	0.0
Employment status^a^												
Full‐time paid work	1700	78.2	187	77.9	196	65.1	93	68.4	88	62.0	117	64.6
Part‐time paid work (including casual paid work, n = 29	177	8.1	16	6.7	23	7.6	11	8.1	11	7.7	25	13.8
Unemployed (including unpaid voluntary work, n = 5)	167	7.71	25	10.4	18	5.9	7	5.1	3	2.1	1	0.6
Student or pupil	43	2	6	2.5	4	1.3	0	0.0	0	0.0	1	0.6
Looking after home/family	21	1	1	0.4	14	4.7	4	2.9	4	2.8	1	0.6
Unable to work due to sickness/disability	14	0.6	0	0.0	3	1.0	0	0.0	2	1.4	2	1.1
Gave up my job after I had my baby	–	–	–	–	5	1.7	7	5.1	4	2.8	4	2.2
Other	43	2	4	1.7	17	5.6	4	2.9	7	4.9	9	5.0
Not reported	9	0.4	1	0.4	21^b^	7.0	10^c^	7.4	23^d^	16.2	2^e^	11.6

a At the time of completing each survey.

b “On paid maternity leave,” n = 15; “Unpaid maternity leave,” n = 3; “Not in paid work or studying at present,” n = 2; Not reported, n = 1.

c “On paid maternity leave,” n = 4; “Unpaid maternity leave,” n = 4; “Not in paid work or studying at present,” n = 2.

d “Unpaid maternity leave,” n = 8; “Not in paid work or studying at present,” n = 14; “Gone back to paid employment,” n = 1 (type of employment not stated).

e “Not in paid work or studying at present,” n = 20; Not reported, n = 1.

### 3.1 Barriers

#### 3.1.1 Antenatally

Three‐quarters of the comments in the antenatal (AN) survey (75%, n = 180) related to some aspect of the survey or the research questions, 33 (18%) of which related to potential barriers to completing the survey. Comments included the survey being “long” or “very long” (n = 17), “personal” or “intimate” (n = 8), “repetitive” (n = 5), or containing questions they found “ambiguous” or “unclear” (n = 3). Other comments related to some questions not being applicable to them because of their personal circumstances, that is, not in a relationship, or being difficult for some women to complete;

Happy to take part. Might be difficult for people for whom English is not their first language, for people who find it difficult to read and write.

[AN‐ID1‐0430]

Twelve women gave reasons for the delay in returning the survey, and these mainly related to being busy or forgetting to post it;

Sorry to reply so late but I do find it difficult to concentrate plus I am working full time which takes 12 h out of my day (including travelling).

[AN‐ID1‐0487]

Sorry for the delay I was extremely busy with work, apologies again.

[AN‐ID1‐1050]

Other women commented on how they were feeling, and these comments illustrated the challenges of and potential barriers to recruiting women to research in early pregnancy;

Sorry for delay ‐ sickness only starting to ease in the last week ‐ 19 weeks tomorrow!

[AN‐ID1‐0157]

Week 16 ‐ feeling very tired to point of exhaustion…5/6 trips a night to toilet first 14 weeks ‐ very little sleep

[AN‐ID1‐0023]

Apologies for delay in completing. I experienced unforeseen events unrelated to pregnancy recently. At early stages of pregnancy I was not confident in pregnancy to complete the form.

[AN‐ID1‐0958]

#### 3.1.2 Postpartum

Eighty‐eight comments in the four postpartum surveys related to delays in returning the survey; 3‐months postpartum [PP3], n = 30; 6‐month postpartum [PP6], n = 14; 9‐month postpartum [PP9], n = 19; and 12‐month postpartum [PP12], n = 25. Reasons for the delayed return included; “moving house” (n = 6); being “out of the country”/”on holiday” (n = 5); “busy” (n = 6); and “forgot” (n = 4). One woman referred to a family member's illness and another, at 12 months postpartum, described herself as “*exhausted.”* Women were not asked to give reasons, but many offered explanations;

I finished very slow [Re returning the survey]…I was 90% ready with it but around the beginning of [month] the baby starts crying during feeding. It was a hard time for me.

[PP3‐ID2‐0714]

Hard to complete [section on labour] ‐ traumatic and I am still very emotional.

[PP3‐ID2‐0318]

Sorry it took me so long to get the survey back to you. It's been hectic with my son and he had a cold, then I did, then he got his injections. Hope I haven't delayed your work too much.

[PP6‐ID3‐0883]

It took me ages to fill it up because my baby does need a lot of attention, so active and doesn't sleep during the day.

[PP9‐ID4‐1047]

One woman gave a detailed explanation of her circumstances;

Just wanted to apologise for taking so long to fill them out. As you know I have postnatal depression and have found this year extremely hard as I never expected to get depressed.

[PP12‐ID5‐1179]

These women's comments illustrate the busyness of women's lives as new mothers and again, point to potential barriers in recruiting women, and then retaining them in research studies.

### 3.2 Benefits

Many women said they benefitted personally by having the opportunity to have their voice heard, from reviewing their own progress as they completed each survey, and from getting information. Questions on various health issues helped many women think about and reflect on their experiences. Four sub‐themes emerged: “access to information,” “relaxation and enjoyment,” “stopping to think” and “prompt to get help.”

### 3.3 Access to information

All of the surveys asked questions about several health problems, for example, urinary or anal incontinence, having pain during sexual intercourse etc., and many women said that the questions alone were a valuable source of health information;

This is a nice way to get information on many things and…I have learnt a lot of things…thanks.

[AN‐ID1‐1926]

I thought this survey was very helpful and the questions were v. good. I think it's great you have numbers to ring for everything to help young mothers in need. [Re inclusion of notices on where to get help for each problem].

[AN‐ID1‐0994]

Others said they would mention the study to their friends;

I've found this process very interesting so far and will check out the website asap. Also I will mention the survey at my breastfeeding support group for any pregnant women who may want to take part (if it is still open). Thank you for your efforts to understand more about these subjects. (I know a friend who had major complications and was doubly incontinent for months, so I know how very distressing it can be).

[PP3‐ID2‐0133]

Some women identified gaps in their knowledge about their health after childbirth;

These surveys have highlighted for me the huge gaps in my knowledge regarding postpartum care for myself. I also have a lot of gaps in my memory of labour and childbirth and now that I have settled into motherhood (as much as one can do at this stage) I have a huge list of unanswered questions. I'm sure with the state of the public health sector at present it is out of the question but I believe a ‘debrief’ (for want of a better word) for mothers who experienced difficult pregnancies/childbirth at a later date (3‐6 months postpartum) would be of a great benefit for parents.

[PP6‐ID3‐2072]

Findings from the study were covered in the national media, and some women welcomed this as a way to raise public awareness about the health problems women can experience;

The recent release and publicity of some of the findings is making me and others realise we are not alone and that we should talk about it and share the less desirable physical issues after having a baby. Irish people still have hang‐ups about sex and our body functions. This study will shine a light and educate women and physicians on what are natural problems that are not taken seriously enough by women and general public.

[PP9‐ID4‐0750]

I find it a very interesting experience to be taking part in the study. I hope our information will be of help to future women. I would like to see some more info given to women on postnatal recovery as I think the mental side effects of what happens to one's body and relationships can be very unexpected for first‐time mothers.

[PP9‐ID4‐1496]

I am delighted to have taken part in this survey. I think it is really important to understand what we as new mums go through! Good work and don't hesitate to contact me!

[PP12‐ID5‐1595]

### 3.4 Relaxation and enjoyment

Women described completing the surveys as relaxing and enjoyable;

This survey relaxed me and took my mind off work + home life for 40 minutes so thank you, and [it] made me have a good think when answering the questions.

[AN‐ID1‐1669]

I enjoyed this survey, seeing the types of questions actually opened my eyes to the types of problems that some women must be experiencing.

[PP3‐ID2‐0793]

It was nice to be asked questions that may not have been put to me by [the] hospital or my GP. It was good to go back over my labour and weeks after baby's birth and answer questions of how I am dealing with being a new mother.

[PP3‐ID2‐2151]

I enjoyed filling out the surveys, for me I found it relaxing because it was about me and my experiences…nobody tells you the ‘after affects’ after you have a baby. Even though my baby was planned, I found that after I had my baby I suffered with depression and I can see I have come a long way since first writing in the survey several months ago and now I almost feel back to normal. Thank you for everything.

[PP9‐ID4‐1996]

### 3.5 Stopping to think

Many women wrote about stopping to think about their health as they completed the surveys;

I found this survey asks some questions where you have to ‘stop & think’ about your answer and think ‘hang on, have I actually felt like this’: I personally feel this is a good thing as my pregnancy is a result of IVF…I would actually think reading about someone experiencing something similar to myself would make me feel better and not alone in what I have experienced or thought.

[AN‐ID1‐1570]

I am enjoying completing the surveys as it makes me think about me and how I have been feeling and coping. To be honest I definitely don't think about me at all as I feel I should concentrate on the baby and making sure my husband doesn't feel left out.

[PP6‐ID3‐0475]

Enjoyed completing the survey, did it in stages over a few days. It became a great talking point between my partner and I…can't describe it really but the questionnaire allows me to stop and think about my own health etc.

[PP6‐ID3‐1904]

I find this survey brilliant, it gives you a good insight into how you are feeling emotionally and physically cause you do not stop and think about how you are really feeling on the inside until you read the questions. I find it satisfying to know I am in good shape and great frame of mind. The surveys are brilliant, can't wait to fill in my next survey.

[PP6‐ID3‐1102]

For some postpartum women, the study was the only time they were asked “*how are you?”*;

This can be the only opportunity I am asked about how “I” am.

[PP9‐ID4‐1723]

I have found there is very little support available after the initial birth, especially for working mums, so it was nice to be asked how I was doing.

[PP12‐ID5‐0608]

### 3.6 Prompt to get help

Completing questions about health problems prompted some women to seek professional help;

I realised by doing this survey about my urinary leakage during the pregnancy and feel a bit worried for the future. I will talk to the physiotherapists at the [hospital] about the problem.

[AN‐ID1‐1962]

I just went to the GP this morning as this survey forces/encourages me to examine how I am feeling, I discussed [listed several health issues]…will call a doctor at the [hospital] to arrange an appointment. I kept putting off visiting the doctor but feel glad I have told her now…need to do my PFE (pelvic floor exercises) again daily, I have gotten out of the habit and need to make time for them again.

[PP6‐ID3‐1091]

I have started to take better care of myself in the last 3 to 4 months to see if I can improve my pelvic situation and I do put that down to this study, so thank you.

[PP9‐ID4‐0475]

I really enjoyed completing the surveys. I found it was all handled really well. It was highlighted to me that things I am not happy with (bowel) I should see someone about. Thank you for asking these questions…I don't feel that people ask how we are post labour, it's all about the baby. The silence makes you think that you must be alone in your worries, but reading your findings I know I am not, I just wish we were talking about it more.

[PP12‐ID5‐2002]

## 4 DISCUSSION

Findings on taking part in research revealed some real and potential barriers and many unanticipated benefits for women. The main barriers related to the survey(s) being long, being too busy as a first‐time mother to complete them on time and questions being intimate or repetitive. While others have identified lack of time or inconvenience as being a barrier to both recruitment and retention to intervention studies or trials,^7^, ^16^ many women in this study completed a lengthy survey with intimate questions during the early stages of pregnancy, despite experiencing sickness or pregnancy‐related worries. Nevertheless, their comments identify potential barriers to recruiting participants in longitudinal studies during pregnancy. The fact that 87% of women completed the survey at 3‐month postpartum, a time when they were busy caring for their first baby, and that 70% of them completed the 12‐month survey, indicates that, once these women had given their commitment, the vast majority continued to participate. Given the high percentage of comments describing positive experiences of taking part in the study and completing surveys, this style of survey may act as an example of how to retain participants in longitudinal studies during and after pregnancy.

Four sub‐themes relating to the benefits gained emerged; “access to information,” “relaxation and enjoyment,” “stopping to think” and “prompt to get help.” Similar to Barret et al's findings^9^ many women said the surveys gave them “access to information,” and that they became informed by completing the surveys. The surveys asked questions about urinary and anal incontinence, pain, sexual and mental health issues and partner violence, and women said they learnt what was/was not normal in relation to one or some of these issues. Access to high‐quality information, including information on specific health conditions/treatments,^17^ enables consumers to participate in their health decisions, increases patient satisfaction, health knowledge, recall of clinical consultations and understanding of the risks and benefits of treatment.^18^, ^19^, ^20^ Trials on decision‐support systems suggest that access to evidence‐based information may reduce healthcare demand,^17^, ^21^ and informed patients may be more likely to make improved healthcare decisions.^22^, ^23^ While healthcare users’ preferred source of health information is a health professional,^24^, ^25^, ^26^ some women taking part in the MAMMI study learnt new information about common health problems simply by taking part, suggesting, perhaps, that their other sources of information may be inadequate/insufficient. We did not ask women specifically if they had heard/read about these health problems elsewhere but these comments, from women of all age groups and education levels, suggest that a considerable number of women have unmet information/education needs postpartum.

Some women were pleased to be asked about how they were feeling, perhaps indicating that taking part gave them a sense that they, and their health, were important, which they had not felt from contacts with healthcare professionals or their social circle. Some of the women interviewed by Baker et al^6^ felt “special” when approached to take part in research, felt proud that they had contributed and important that their views were acknowledged. Although we cannot elaborate on what women in our study meant by being pleased to be asked about their experiences, contributing to research may have given them a sense of pride in that they were contributing to or advancing knowledge. Many women said completing the surveys was enjoyable and relaxing. For some, the survey took their mind off work and home life for a short time while, for others, it was “*nice to be asked questions that may not have been put to me by* [the] *hospital or my GP.”* Findings from other studies have shown that many healthcare users are dissatisfied with the quality of the interactions with their healthcare provider,^27^, ^28^, ^29^ with some communication problems attributable to healthcare providers’ focus on diseases and their management, rather than on the people, their lives and their health issues. Healthcare professionals can sometimes lack time or confidence in discussing sensitive issues, such as contraception^30^ and sexual health,^31^ with clients. In this study, women gained access to information simply from reading the survey questions, and from the notices of where to get help/support related to specific health problems in each survey. Women could also access information via the *Sharing the findings* section of the MAMMI study website (http://www.mammi.i.e/findings.php) and could benchmark their own health against the overall results. Being asked questions not asked at the hospital or by their general practitioner (GP) was described as eye‐opening. Many questions asked about sensitive and intimate issues, for example, leaking urine, pain during sexual intercourse, intimate partner violence, yet women described completing the surveys as relaxing and enjoyable. This may suggest that access to information enhanced their sense of control over how they managed their health issues, and relieved anxiety.^32^

Exposure to stress and adversity is acknowledged as having a negative influence on the developing foetus and child,^33^, ^34^, ^35^, ^36^ and the effect of information‐giving on levels of pain experienced are well known.^37^ In other healthcare areas, such as oncology and surgery, there is evidence to suggest that giving patients information on the procedure they are about to undergo can significantly reduce their emotional distress and improve their psychological and physical recovery.^38^, ^39^ In our study, women said the surveys gave them time to reflect on their health and life during the first year of first‐time motherhood, see how much progress they had made and be satisfied knowing they were in good health. The questions on each morbidity made them “stop and think” about how they really felt. For others, they helped them realize they had a health problem, and prompted them to take better care of themselves or seek professional help, even up to 12 months postpartum. Contrary to previously held beliefs that women's bodies return to their pre‐pregnant state within 6 weeks postpartum, findings from this and other studies show that some health problems are present before women's first pregnancy,^10^, ^40^ that considerable proportions of women experience persistent and long‐term morbidities^41^, ^42^, ^43^, ^44^, ^45^ and that some women's health can deteriorate during the year after giving birth. A systematic review of morbidities experienced by women who had a postpartum haemorrhage found that women who suffered problems such as coagulopathy [a disorder affecting the blood's ability to clot] (1.74 %), post‐traumatic stress disorder (3%), or required readmission to hospital 1‐3 months postpartum (3.6 %) described their health as “much worse than 1 year ago” (6%).^46^ Women having caesarean section, in particular, have a higher readmission to hospital rate (4.33%)^47^ and may suffer continuing morbidity in the first year postpartum.

Our findings add to those of previous studies in terms of research participants benefitting by gaining access to information. We also identified new information in relation to first‐time mothers benefitting by “stopping to think” about their personal experience and taking time to reflect on their own health, and these findings may contribute to initiatives aimed at improving postpartum care.^48^ An important new finding is that taking part in this cohort study enabled women to identify that they had a health problem, which prompted them to take better care of themselves and/or seek professional help.

### 4.1 Implications

While it was gratifying that so many women benefitted simply from taking part, it is concerning that the survey content gave others information, for the first time, about common postpartum health problems. The inclusion of notices of sources of support on where to get help/advice for particular health problems was clearly of benefit to many. These findings have both practice and research implications; from a clinical practice perspective, it points to deficits in the way information is provided within maternity care. For researchers, it highlights the type and amount of information that should be given to childbearing women participating in research.

### 4.2 Limitations

Findings are based on comments from first‐time mothers who were taking part in a longitudinal maternal health study and are not necessarily transferrable to non‐pregnant populations. Fewer women with no formal or lower secondary education commented in any survey, which limits our findings. However, findings in relation to learning new information, being offered contact details of sources of help and being prompted to seek professional help may be applicable to all women.

## 5 CONCLUSION

Women used the free‐text section on the final survey page to say how they benefitted from taking part in the MAMMI study, or the difficulties they had experienced. As they completed the surveys, women learnt new information and took time to reflect on their health. The survey content encouraged some women to take better care of themselves and prompted others to seek professional help. If taking part in research can have such positive benefits, all researchers should find ways of making health information more available through surveys and other research tools and, in this way, enable participants to benefit personally from taking part.

## CONFLICT OF INTEREST

The authors declare they have no conflict of interest.
