# Supplementary material for: Stop, think, reflect, realize—first‐time mothers’ views on taking part in longitudinal maternal health research
Source: Health Expect. 2019 Feb 21;22(3):415–25. doi: 10.1111/hex.12861 (PMC6543136; doi:10.1111/hex.12861)
Supplement: Supplementary file 1 [file HEX-22-415-s001.docx]

| **Table 1 Participants’ characteristics compared with the national statistics** | | | | | | |
| --- | --- | --- | --- | --- | --- | --- |
|  | **The MAMMI Study**  **n=2174** | | **National Perinatal Reporting System (NPRS) 2015**  **n=67,610** | | **Central Statistics Office (CSO) 2011**  **n=1,019,091** | |
|  | n | % | n | % | n | % |
| **Nationality** |  |  |  |  |  |  |
| Irish | 1557 | 71.6 | 52571 | 77.8 | 809847 | 79.5 |
| Irish traveller | 2 | 0.1 | Reported by country/continent | | 6022 | 0.6 |
| African | 23 | 1.1 |  |  | 14646 | 1.4 |
| Chinese | 11 | 0.5 |  |  | 6572 | 0.6 |
| Any other white background | 506 | 23.3 |  |  | 135945 | 13.4 |
| Any other black background | 3 | 0.1 |  |  | 1400 | 0.1 |
| Any other Asian background | 42 | 1.9 |  |  | 19739 | 1.9 |
| Other, including mixed background | 20 | 0.9 |  |  | 14268 | 1.4 |
| Not stated | 10 | 0.5 | 235 | 0.3 | 10652 | 1.1 |
|  |  |  |  |  |  |  |
| **Age group** |  |  |  |  |  |  |
| 18 to 24 years | 177 | 8.1 | 3478[^†^](https://en.wikipedia.org/wiki/Dagger_(typography)) | 14.1 | - | - |
| 25 to 29 years | 471 | 21.7 | 5593 | 22.6 | - | - |
| 30 to 34 years | 941 | 43.3 | 9083 | 38.8 | - | - |
| 35 to 39 years | 484 | 22.3 | 4479 | 18.1 | - | - |
| Over 40 years | 94 | 4.3 | 998[^‡^](https://en.wikipedia.org/wiki/Double_dagger_(typography)) | 4.0 | - | - |
| Not stated | 7 | 0.3 | - | - | - | - |
|  |  |  |  | |  |  |
| **Relationship status** |  |  |  | |  |  |
| Married | 1312 | 60.3 | 40968 | 62.2 | - | - |
| Living with partner | 575 | 27.3 | 23990 | 36.4 |  |  |
| In a relationship - not living together | 160 | 7.4 |  |  |  |  |
| Single | 79 | 3.6 |  |  | - | - |
| Divorced, separated | 4 | 0.2 | 830 | 1.3 | - | - |
| Widowed | 1 | 0.1 | 63 | 0.1 | - | - |
| Other | 17 | 0.8 | - | - | - | - |
| Not stated | 6 | 0.3 | - | 0.0 | - | - |
| **BMI category** *(*[^§^](https://en.wikipedia.org/wiki/Section_sign)*National data not available – data from one site hospital 2015)* |  |  |  | |  | |
| Underweight (≤18.49kg/m^2^) | 103 | 4.7 | 168[^§^](https://en.wikipedia.org/wiki/Section_sign) | 2.0 | Not reported | |
| Normal (18.5-24.99kg/m^2^) | 1320 | 60.7 | 4454[^§^](https://en.wikipedia.org/wiki/Section_sign) | 53.3 | - | - |
| Overweight (25-29.99kg/m^2^) | 375 | 17.2 | 2323[^§^](https://en.wikipedia.org/wiki/Section_sign) | 27.8 | - | - |
| Obese (30-34.99kg/m^2^) | 159 | 7.3 | 838[^§^](https://en.wikipedia.org/wiki/Section_sign) | 10.0 | - | - |
| Very obese (≥35kg/m^2^) | 38 | 1.7 | 410[^§^](https://en.wikipedia.org/wiki/Section_sign) | 4.9 |  |  |
| Not reported | 179 | 8.2 | 168[^§^](https://en.wikipedia.org/wiki/Section_sign) | 2.0 |  |  |
| **Highest education attainment** |  |  |  |  |  |  |
| No formal - lower secondary | 51 | 2.4 | Not reported | | - | - |
| Upper secondary, apprenticeship/vocational | 488 | 22.5 | - | - | - | - |
| National certificate/Diploma, Institute of Technology or equivalent | 205 | 9.4 | - | - | - | - |
| Primary degree/professional qualification of degree status/ postgraduate certificate or diploma | 952 | 43.8 | - | - | - | 55.3[^¶^](https://en.wikipedia.org/wiki/Pilcrow) |
| Postgraduate degree/PhD | 464 | 21.3 | - | - | - |  |
| Not stated | 14 | 0.6 | - | - |  |  |
| **Employment status** |  |  |  |  |  |  |
| Full time paid work | 1700 | 78.2 | Classification according to occupation and socio-economic group | | Reported as persons in the labour force as a proportion of the population | |
| Part time paid work | 148 | 6.8 |  |  |  |  |
| Casual paid work | 29 | 1.3 |  |  |  |  |
| Looking for first job | 8 | 0.4 |  |  |  |  |
| Unemployed | 154 | 7.1 |  |  |  |  |
| Student or pupil | 43 | 2.0 |  |  |  |  |
| Looking after home/family | 21 | 1.0 |  |  |  |  |
| Unable to work due to sickness/disability | 14 | 0.6 |  |  |  |  |
| Unpaid voluntary work | 5 | 0.2 |  |  |  |  |
| Other | 43 | 2.0 |  |  |  |  |
| Not reported | 9 | 0.4 |  |  |  |  |
| Healthcare Pricing Office (HPO) (2017) National Perinatal Reporting System (NPRS) *Perinatal Statistics report 2015.* Available at: [*http://www.hpo.ie/latest_hipe_nprs_reports/NPRS_2015/Perinatal_Statistics_Report_2015.pdf*](http://www.hpo.ie/latest_hipe_nprs_reports/NPRS_2015/Perinatal_Statistics_Report_2015.pdf)  Central Statistics Office (CS0) (2011). Available from: [*https://www.cso.ie/en/census/census2011reports/census2011profile7religionethnicityandirishtravellers-ethnicandculturalbackgroundinireland/*](https://www.cso.ie/en/census/census2011reports/census2011profile7religionethnicityandirishtravellers-ethnicandculturalbackgroundinireland/)*.* Percentages calculated from data in Table CD795 *Population Usually Resident and Present in the State by Ethnic or Cultural Background, Regional Authority, Age Group and Gender.*  [^†^](https://en.wikipedia.org/wiki/Dagger_(typography))Women aged 20-24 years; women aged less than 20 years reported separately (n=1068, 4.3%).  [^‡^](https://en.wikipedia.org/wiki/Double_dagger_(typography))Number calculated from total number of maternities to primiparous women.  [^§^](https://en.wikipedia.org/wiki/Section_sign)Includes primiparous and multiparous women, and calculations based on women’s weight and height at first booking visit during pregnancy (Coulter-Smith, S. (2016) The Rotunda Hospital Clinical Report 2015). *Available at:* [*https://rotunda.ie/rotunda-pdfs/Clinical%20Reports/Clinical%20Report%202015.pdf*](https://rotunda.ie/rotunda-pdfs/Clinical%20Reports/Clinical%20Report%202015.pdf)  [^¶^](https://en.wikipedia.org/wiki/Pilcrow)Proportion of women with third level qualification in Ireland in 2013. Central Statistics Office (CS0) (2013) *Women and Men in Ireland.* Available at: [*https://www.cso.ie/en/releasesandpublications/ep/p-wamii/womenandmeninireland2013/educationlist/education/*](https://www.cso.ie/en/releasesandpublications/ep/p-wamii/womenandmeninireland2013/educationlist/education/) | | | | | | |
